# Latent persistence in fMRI dynamics during human memory consolidation

**DOI:** 10.1016/j.ynirp.2026.100398

**Published:** 2026-08-17

**Authors:** Maria Bartzioka, Mohammad Khosravi

**Affiliations:** Delft Center for Systems and Control, Delft University of Technology, Mekelweg 2, Delft, 2628 CD, the Netherlands

**Keywords:** Memory consolidation, Koopman operator, fMRI, Dynamic mode decomposition, Systems neuroscience

## Abstract

Human memory consolidation involves the gradual stabilization and reorganization of memory traces over time. Despite numerous empirical and computational accounts emphasizing different aspects of this process, an integrated framework for evaluating consolidation theories against brain data remains limited. We propose a biologically informed, data-driven framework based on Koopman operator analysis to examine latent dynamical structure in fMRI signals associated with memory consolidation. The Koopman framework lifts nonlinear brain dynamics into a linear function space, enabling spectral characterization of persistence and stability. In practice, we employ Dynamic Mode Decomposition (DMD) together with an observability-aware extension tailored to consolidation-related neural dynamics. We organize existing theories into three functional clusters: standard consolidation, episodic replay during rest, and distributed long-term storage, and then align open-access fMRI datasets with each cluster to assess their dynamical plausibility. Across datasets, delayed or repeated retrieval conditions generally tend to show greater spectral persistence than early encoding-related conditions. Among the three analyses, Cluster 3 yielded the clearest statistically reliable subject-level contrast, with semantically abstracted autobiographical content exhibiting higher mean eigenvalue magnitude and more near-unit modes than event-specific episodic content. This finding is compatible with transformation-oriented and distributed-storage accounts but does not constitute a direct temporal test of consolidation. Replay-related conditions show strong spectral differentiation across task states, although part of this separation likely reflects task structure in addition to consolidation-related dynamics. For the standard consolidation cluster, effects are directionally consistent with theory but remain small and not statistically significant at the subject level. Overall, the proposed framework provides an interpretable operator-theoretic approach for linking memory consolidation theory to latent brain dynamics and for comparing competing accounts in a common spectral language.

## Highlights

• Memory consolidation exhibits cluster-dependent latent dynamical stability profiles.
• Koopman spectral analysis differentiates persistence structure across encoding, retrieval, and post-encoding conditions.
• Distributed long-term storage yields the clearest subject-level contrast, with more semanticized autobiographical content showing greater latent persistence than event-specific episodic content.
• Replay-related conditions show strong spectral differentiation, part of which likely reflects task structure.
• The proposed operator-theoretic framework links consolidation theories to interpretable latent dynamical signatures.

## Graphical abstract

**Figure:**
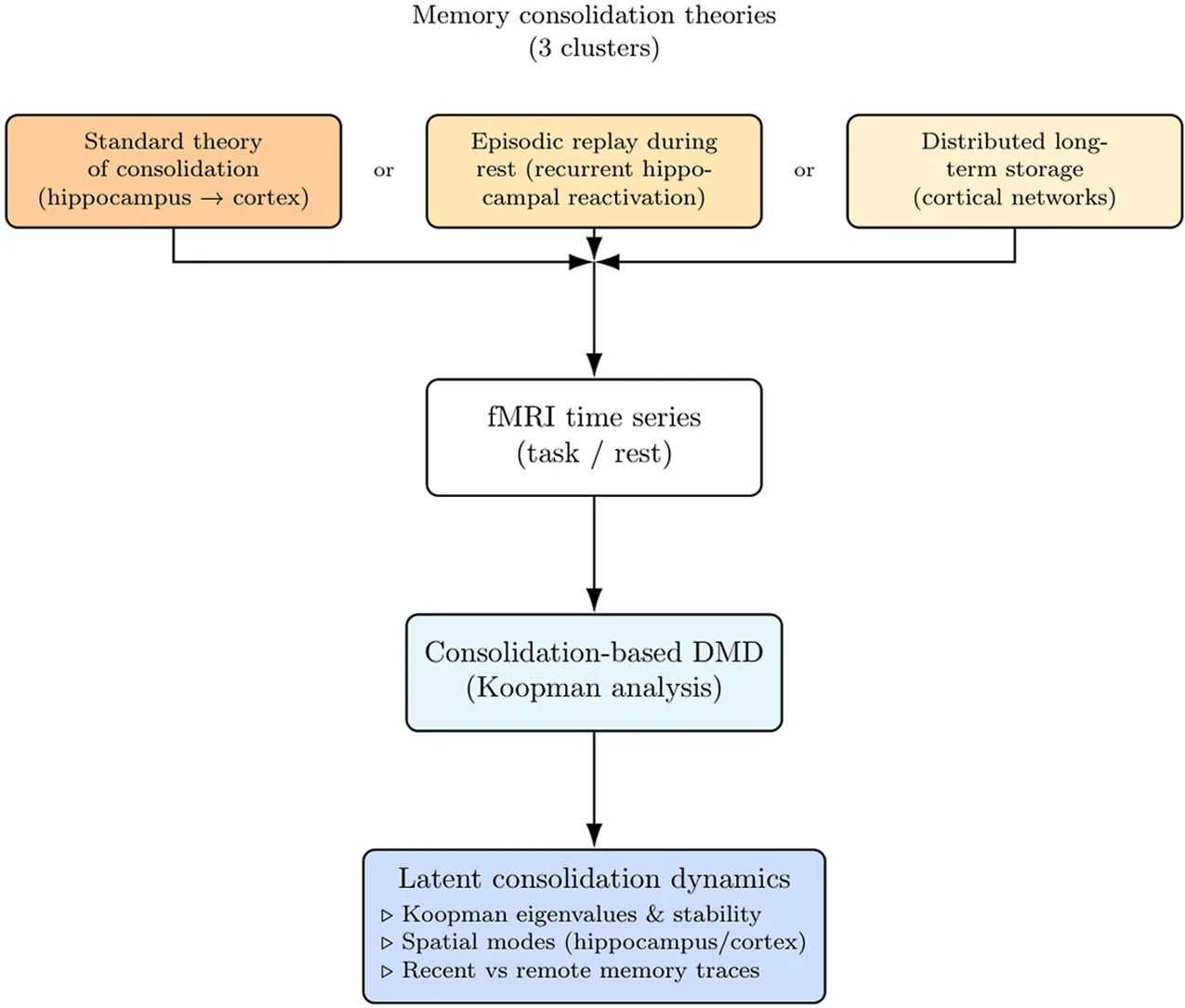

## 1 Introduction

Human memory is a complex biological process involving different brain regions and operating across various temporal and functional scales. Having access to past experiences and knowledge is essential for guiding present behavior, whether performing routine tasks critical for survival, recognizing danger, or engaging in complex decision-making. Memory is typically categorized into distinct modules based on temporal duration: sensory memory, short-term memory, and long-term memory. Sensory memory briefly retains incoming stimuli, short-term memory maintains information over seconds to minutes, while long-term memory supports the storage and retrieval of information over extended periods, potentially lasting a lifetime. This information ranges from facts and skills to experiential knowledge, accessed either consciously or unconsciously when required (Baddeley, 1999). In this work, we focus on long-term memory consolidation: the process by which newly formed, fragile memory traces are stabilized and integrated over time. Due to the complexity of this process, a unified model capturing its diverse biological and functional aspects remains elusive.

Reflecting the complexity of memory consolidation, a wide range of empirical and computational models have been proposed, including the Standard Model (Squire and Alvarez, 1995), the Reconsolidation Theory (Nader and Hardt, 2009), and various network-based approaches. Each model emphasizes different mechanisms, such as systems-level memory transfer, reactivation dynamics, retrieval-based stabilization, or molecular processes underlying synaptic change. However, a unified framework that integrates these diverse perspectives and that also captures the core principles of consolidation remains absent. This is largely due to the difficulty of validating theories against empirical brain data, given the latent, distributed, and nonlinear nature of the process of memory consolidation. Furthermore, existing computational approaches often abstract away from neurobiological detail and lack the capacity to extract interpretable, time-dependent dynamics, reinforcing the need for a biologically informed modeling framework grounded in real data.

To address this challenge, we propose a biologically informed, data-driven framework based on Koopman operator theory (Koopman, 1931), a spectral operator-theoretic framework that represents nonlinear dynamical systems through linear evolution in a lifted function space (Mezić, 2005; Budišić et al., 2012; Mauroy et al., 2020). This approach transforms nonlinear brain dynamics into a linear representation in the function space, allowing for spectral decomposition into eigenvalues, eigenfunctions, and modes. These triplets offer insights into latent neural states and their evolution over time, features central in understanding memory consolidation. In the absence of explicit dynamical models, we apply Dynamic Mode Decomposition (DMD) to approximate the Koopman operator directly from fMRI time series data. To capture the consolidation-specific structure, we introduce a biologically guided variant of DMD. We organize existing consolidation theories into three functional clusters.

(1) standard consolidation, predicting increased stability with memory age;
(2) episodic replay during rest, predicting transient reactivation-related dynamics; and
(3) distributed long-term storage, predicting persistent multi-regional representations. For each cluster, we derive explicit spectral predictions and evaluate them using open-access fMRI datasets aligned to their core assumptions.

Specifically, we test the following hypotheses.

• **H1:** Remote memory retrieval will exhibit higher mean eigenvalue magnitudes and increased near-unit eigenmodes compared to recent retrieval, consistent with standard consolidation.
• **H2:** Resting-state intervals following encoding will display transient or weakly persistent spectral modes consistent with replay-related dynamics.
• **H3:** Distributed long-term storage will manifest as persistent, multi-regional Koopman modes spanning hippocampal and neocortical ROIs.

Our contributions are threefold.

1. First, we propose a dynamical clustering of consolidation theories based on their predicted stability profiles.
2. Second, we introduce a biologically grounded Koopman spectral framework for extracting latent stability signatures from neuroimaging data.
3. Third, we show that near-unit Koopman eigenmodes provide an interpretable marker of latent persistence, with the clearest subject-level support arising for the distributed-storage cluster and more limited evidence for the standard and replay-related clusters.

By framing consolidation as a problem of latent dynamical stability, our work establishes operator-theoretic spectral analysis as a principled bridge between cognitive theory and large-scale brain dynamics.

The remainder of this paper is organized as follows: Section 2 reviews empirical and computational models of memory consolidation and introduces a conceptual classification via shared theoretical principles. Section 3 presents the methods and mathematical foundation of our approach (Koopman operator theory and DMD) and our consolidation-informed DMD extension. Initially the dataset selection criteria, preprocessing and ROI extraction are detailed. Section 3 is concluded with the analysis pipeline for extracting latent dynamical signatures. Section 4 presents the results obtained for each cluster and concludes with a cross-cluster comparison. Section 5 discusses challenges, research gaps as well as directions for future work. Finally, Section 6 concludes the paper.

## 2 Conceptual clustering of memory consolidation approaches

Memory consolidation operates across multiple spatial and temporal scales, from synaptic plasticity at the cellular level to distributed interactions between hippocampal and neocortical networks (Baddeley, 1999; Squire et al., 2015). While mechanistic descriptions span microscale neuron models, mesoscale network dynamics, and macroscale neural mass formulations (Betzel and Bassett, 2017; Siettos and Starke, 2016), systems-level consolidation specifically concerns the long-term reorganization of large-scale brain networks. Here, we focus on this systems-level perspective and review the dominant theoretical accounts that aim to explain how memory traces stabilize over time.

### 2.1 Empirical models of systems consolidation

Memory consolidation refers to the process by which newly acquired information is stabilized over time, enabling long-term storage and recall. Originally proposed by (Müller and Pilzecker, 1900), consolidation is now recognized as a multi-faceted process involving both synaptic and systems-level mechanisms driven by neural plasticity. Synaptic consolidation occurs rapidly, within seconds to hours, at the cellular level, whereas systems consolidation unfolds gradually over days to years, involving the reorganization of brain networks, particularly those centered on the hippocampus. Throughout this paper, the term *consolidation* will specifically refer to systems-level consolidation.

The Standard Model posits that memory initially relies heavily on the hippocampus and gradually becomes stabilized in the neocortex through synaptic plasticity and evolving network connectivity (Squire and Alvarez, 1995; Squire et al., 2015; Frankland and Bontempi, 2005). Similarly, the Two-Stage Model (Dudai, 2004) emphasizes the temporal distinction between rapid synaptic consolidation and the slower systems-level reorganization that supports long-term memory storage. Extending these frameworks (Remme et al., 2021), propose a circuit-level mechanism whereby Hebbian plasticity in parallel synaptic pathways enables the gradual transfer of memory traces from hippocampal to neocortical circuits.

Although this general consolidation framework is widely accepted, various empirical models have been proposed to refine or challenge its assumptions by emphasizing additional mechanisms. One such model is Reconsolidation Theory (Nader and Hardt, 2009), which highlights the dynamic nature of memory by proposing that even stabilized memories can re-enter a transiently labile state upon retrieval. These reactivated traces require reconsolidation to persist, engaging molecular cascades similar to those involved in initial consolidation. Building on this dynamic perspective (Fiebig and Lansner, 2014), introduce a three-stage network model that spans multiple time scales, from prefrontal working memory to long-term neocortical storage. A key innovation of this model is the implementation of *autonomous reinstatement dynamics*, intrinsic neural replay events that stochastically reactivate stored memory patterns without relying solely on external cues.

Another influential alternative is Multiple Trace Theory (MTT) (Nadel and Moscovitch, 1997), which challenges the notion that hippocampal involvement ceases over time. Instead, MTT posits that the hippocampus remains permanently involved in the retrieval of detailed episodic memories, while the extraction of semantic content over repeated retrievals enables its storage independently in neocortical networks. Supporting this view (Nadel et al., 2007), review evidence from neuroimaging, neuropsychology, and animal studies, emphasizing that episodic memories remain dependent on the hippocampus, whereas semantic memories gradually become neocortically represented. They further highlight prospective memory as a promising domain for differentiating between these memory types.

Expanding on MTT, the Transformation Hypothesis (Winocur and Moscovitch, 2011) proposes that over time, memories shift from context-rich, hippocampus-dependent episodic representations into more abstract, context-poor semantic versions stored in the neocortex. Similarly, the Complementary Learning Systems (CLS) Theory (McClelland, McNaughton, & O'Reilly, 1995) suggests that the hippocampus supports rapid learning of specific experiences, while the neocortex integrates structured knowledge more slowly through interleaved learning. This framework explains why systems consolidation is gradual and why new memories initially depend on the hippocampus before being integrated into neocortical networks.

Finally, experimental work (Ryan et al., 2015) provides causal evidence from animal models that memory traces can remain intact yet inaccessible under retrograde amnesia. Using optogenetics to reactivate engram cells, they restored memory expression despite amnesia, challenging purely storage-based models like the Standard one. This supports retrieval-based perspectives that view memory accessibility as a dynamic process influenced by both neural activation states and network integrity.

### 2.2 Computational formulations

Various attempts have been made to mathematically model the stabilization of long-term memory, ranging from network models to data-driven approaches, reflecting both the complexity of memory and the need for coherent computational frameworks grounded in established theories. One foundational contribution is the Simple Network Model (Alvarez and Squire, 1994), which conceptualizes the Medial Temporal Lobe (MTL) as a regulator that facilitates the gradual transfer of memory traces from hippocampal to cortical regions. Expanding this perspective (Murre et al., 2013), introduce a formal mathematical model describing forgetting and retention dynamics over time. Similarly, multi-stage network models have been proposed, such as the memory partitioning model (Roxin and Fusi, 2013), which offers a systems-level rationale for gradually stabilizing memory across hierarchically organized stores. At the synaptic level (Benna and Fusi, 2016), addresses the plasticity-stability trade-off, proposing a computational mechanism that optimizes memory retention by balancing fast and slow synaptic processes.

Moving toward models that emphasize the dynamic reactivation and updating of memory traces (Fiebig and Lansner, 2014), propose a three-stage network model incorporating autonomous replay mechanisms that operate without external cues. Complementing this systems-level view, the Synaptic Reentry Reinforcement (SRR) model (Wittenberg et al., 2002) highlights the role of N-Methyl-D-Aspartate (NMDA) receptor-dependent plasticity, showing how repeated reactivation stabilizes memory traces over time. Supporting this mechanism (Shimizu et al., 2000), provide a synaptic-level explanation for the role of reactivation in consolidating long-term memories.

Finally, computational models also address the abstraction and transformation of memory traces over time. Building on Complementary Learning Systems (CLS) theory, (McClelland, 2013) demonstrates through simulations that schema-consistent information can be rapidly integrated into neocortical structures without disrupting existing knowledge. This refinement enhances the biological plausibility of CLS by emphasizing the role of prior knowledge in modulating learning rates.

Overall, while these computational models provide valuable insights into memory dynamics, they are often either overly detailed, too abstract for empirical validation, or constrained by their theoretical assumptions. None fully capture the latent, time-evolving nature of consolidation in a way that is both biologically grounded and computationally tractable. This gap motivates the need for a novel, integrative framework. Next, we categorize these models via their shared theoretical foundations.

### 2.3 Clustering by dynamical principles

In this subsection, we propose a novel classification of the existing models of memory consolidation (both empirical and computational) presented in the previous subsection. As our goal is to assess which of these models and their underlying mechanisms can be validated through data, we organize them according to their shared biological principles.

Specifically, based on the neural mechanisms proposed to underlie the stabilization of long-term memory, three major clusters, summarized in the table that follows, emerge.

• **Standard Theory of Consolidation**: Memory traces gradually become hippocampus-independent and solely depend on the neocortex.
• **Episodic Replay During Rest**: Memory traces are dynamic, and retrieval can make them undergo cycles of destabilization and restabilization (reconsolidation).
• **Distributed Long-Term Storage**: Initially encoded context-rich memory traces gradually become context-poor/abstract (transformation), where retrieval creates new hippocampal traces, enriching cortical representations and creating overlapping hippocampal-cortical traces (multiple trace). The repeated creation of hippocampal-cortical interactions supports gradual abstraction of the memory trace (complementary learning systems).

**Fig. 1:**
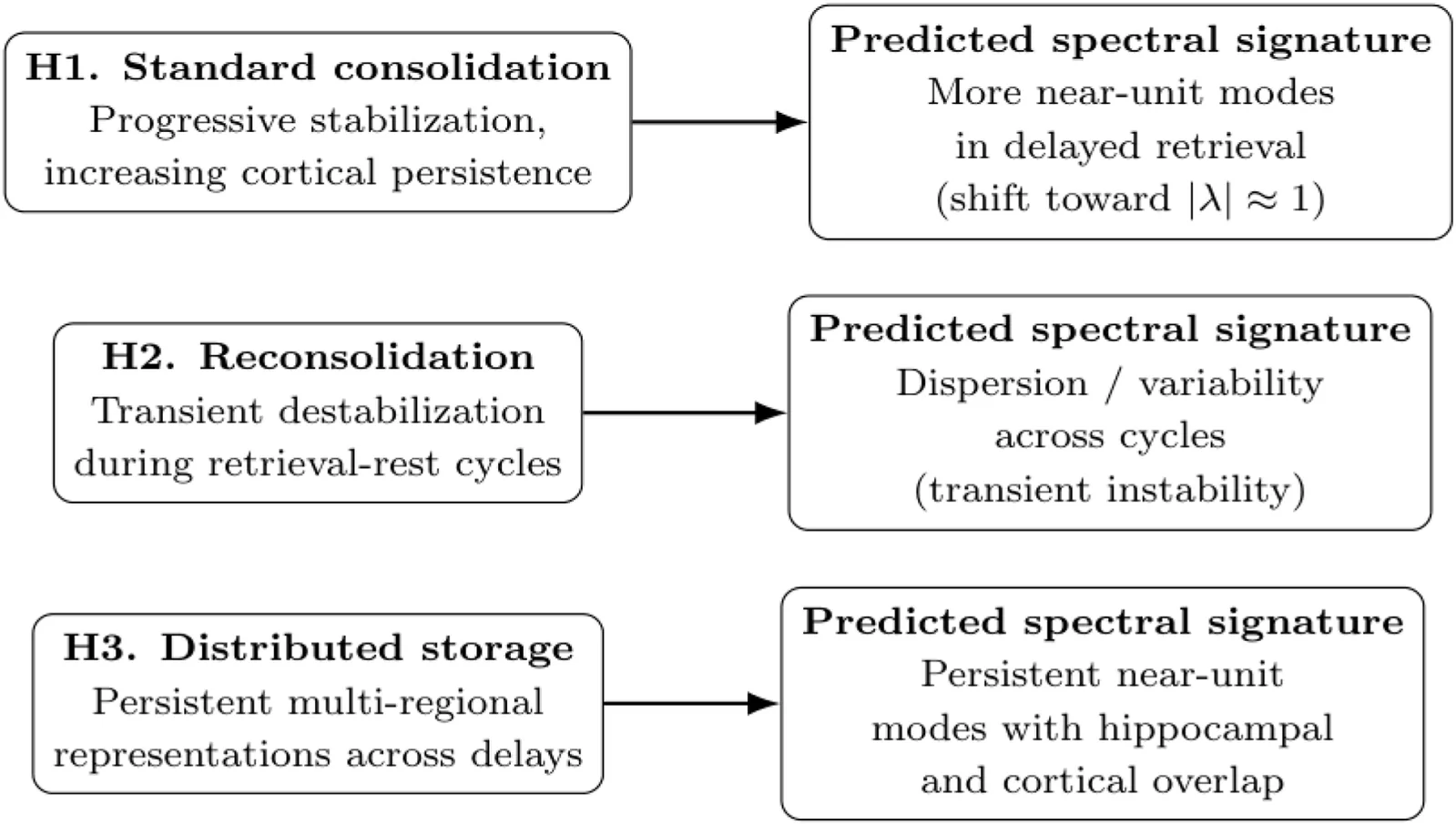
Conceptual mapping from consolidation clusters (H1–H3) to predicted Koopman spectral stability fingerprints. Each hypothesis is evaluated using eigenvalue magnitude structure and spatial mode composition.

The aforementioned clusters differ not merely in anatomical emphasis but in their predicted dynamical fingerprints. More specifically, the difference involves whether memory representations become increasingly stable, undergo transient instability, or remain persistently distributed. This dynamical distinction forms the basis for the spectral evaluation framework that will be introduced in the following section. From a systems-theoretic perspective, each cluster can be interpreted as positing a characteristic spectral signature in the underlying neural dynamics. In particular, consolidation mechanisms may differ in the degree of latent mode persistence, transient variability, or redistribution of dynamical dominance across regions. These differences are not merely conceptual but can be formalized through the spectral properties of a Koopman operator approximation. Accordingly, we reformulate each theoretical cluster as a hypothesis about the expected Koopman spectral structure. Fig. 1 illustrates this translation from biological mechanism to operator-theoretic prediction, explicitly linking consolidation principles to eigenvalue stability and spatial mode composition.

## 3 Methods

In this section, we describe the methodological framework used to evaluate the three consolidation clusters. We first define hypothesis-driven criteria for aligning open-access fMRI datasets with the theoretical assumptions of each cluster. We then detail the preprocessing pipeline and ROI-level time-series extraction applied uniformly across datasets. Finally, we introduce the Koopman-based spectral analysis used to quantify latent dynamical stability. This methodological framework enables an objective comparison of cluster-specific predictions, with results presented in Section 4.

### 3.1 Dataset selection

To evaluate the three consolidation clusters in a principled manner, we adopted a hypothesis-driven dataset selection strategy. Datasets were chosen based on their alignment with the core dynamical assumptions of each theoretical cluster rather than on prior empirical outcomes. This approach ensures that subsequent Koopman-based analyses remain interpretable, comparable, and theory-grounded. The following principles guided dataset inclusion.

1. *Data quality:* Datasets were required to follow established neuroimaging preprocessing standards (e.g., motion correction, slice timing correction, normalization, smoothing) and to be openly accessible under FAIR (Findable, Accessible, Interoperable, Reusable) principles. Adequate sample sizes (approximately ≥20 subjects per group) were required to ensure statistical robustness and generalizability.
2. *Theoretical alignment:* Dataset selection prioritized experimental designs capable of directly testing cluster-specific predictions. Selected datasets included clearly defined tasks contrasting consolidation-relevant stages (e.g., encoding, early retrieval, delayed retrieval, reactivation during rest). Cluster-specific criteria were as follows:

• *Standard consolidation:* Clear separation between early hippocampus-dependent and delayed cortical-dependent retrieval.
• *Episodic replay during rest:* Retrieval paradigms interleaved with rest intervals enabling assessment of reactivation-related dynamics.
• *Distributed long-term storage:* Repeated retrieval across extended delays, ideally contrasting context-rich and context-abstracted representations.

3. *Spatiotemporal resolution:* Datasets were required to provide sufficient spatial coverage of key memory-related regions (hippocampus, medial prefrontal cortex, lateral temporal and parietal cortices) and adequate temporal resolution (ideally TR≤2 s) to capture relevant neural dynamics.
4. *Control conditions:* Appropriate baseline or control conditions were required to isolate consolidation-related processes from non-specific perceptual, attentional, or motor effects.
5. *Behavioral validation:* Behavioral measures, such as accuracy, reaction time, or confidence, are required to support the interpretation of the corresponding neural contrast:

• *Standard consolidation:* We do not require memory performance to improve across delays. Rather, we require that recent and remote conditions show comparable or sufficiently preserved retrieval performance, so that differences in latent dynamical stability will not be confounded by gross differences in retrieval success, forgetting, or task difficulty.
• *Episodic replay during rest:* Performance fluctuations associated with reactivation phases.
• *Distributed storage:* Gradual transformation or abstraction of memory representations.

Guided by these criteria, the following open-access datasets were selected.

• *Cluster 1 (Standard consolidation):* ds005581 (Tompary and Davachi, 2024), which includes encoding versus delayed retrieval contrasts capturing hippocampal–cortical dynamics.
• *Cluster 2 (Episodic replay during rest):* ds003721 (Visser et al., 2021), which includes repeated retrieval interleaved with rest periods to probe reactivation dynamics.
• *Cluster 3 (Distributed long-term storage):* ds003511 (Gilmore et al., 2021), which includes repeated retrieval across extended delays to assess gradual abstraction and distributed representations.

To facilitate interpretation of the cluster-specific analyses, Table 2 summarizes the experimental design, temporal structure, relevant conditions, and primary contrast for each dataset, while Table 1 summarizes the alignment of the included models with their corresponding theoretical frameworks. Together these overviews provide the necessary methodological context for the results presented in the following sections.

**Table 1:** Alignment of each model with the corresponding theoretical framework.

Cluster	Empirical Models	Computational Models
Standard Theory of Consolidation	Standard, Two-Stage	Simple Network, Multi-Stage Partitioning, Synaptic Plasticity-Stability
Episodic Replay During Rest	Reconsolidation, Replay/Reinstatement	Reactivation Network, SRR
Distributed Long Term Storage	MTT, CLS	CLS

**Table 2:** Summary of the datasets and experimental contrasts used for the cluster-specific Koopman/DMD analyses.

Dataset	Memory account	Experimental design and timing	Conditions used in the analysis	Primary contrast
ds005581	Standard consolidation/integration	Object sequences with overlapping structure were learned in two sessions separated by approximately 24 h. Day 1 sequences were treated as remote and Day 2 sequences as recent. Task and resting-state scans were analyzed separately.	Recent and remote task-related conditions; recent and remote post-learning resting-state conditions where available.	Remote versus recent task dynamics, with resting-state contrasts reported separately.
ds003721	Episodic replay during rest	Six approximately 1-min distressing film clips were presented, each followed by 5 min of post-encoding rest. An additional 6-min post-encoding resting-state run was acquired, followed by a cue-guided reactivation session approximately 24 h later.	Film: distressing-film encoding; BI: post-encoding rest with button indication of spontaneous film-related images; React: delayed cue-guided reactivation of selected film memories.	Spectral differentiation across Film, BI, and React.
ds003511	Distributed storage/representational transformation	Overt autobiographical recall of events from earlier the same day, 6–18 months earlier, and 5–10 years earlier. Recalled content was classified by detail type.	Internal details: event-specific episodic content; External details: semantic, generalized, repeated, or non-event-specific content.	External versus Internal detail-related dynamics.

This structured selection strategy ensures that spectral stability metrics can be directly interpreted in light of cluster-specific theoretical predictions.

### 3.2 Preprocessing and ROI extraction

All datasets were preprocessed in MATLAB using SPM12 (Friston, 2007) following a minimal and standardized pipeline designed to preserve temporal structure and inter-regional covariance for subsequent operator-theoretic modeling. Preprocessing was applied directly to BIDS-formatted datasets using custom MATLAB scripts with identical logic across studies; only minimal dataset-specific adjustments were introduced when required (e.g., multi-echo acquisitions).

*Anatomical preprocessing.* For each subject (and session, when applicable), the T1-weighted anatomical image was processed using SPM12's unified segmentation routine to estimate the nonlinear deformation field mapping native space to MNI space. To ensure reproducibility and reduce storage redundancy, only the deformation fields were retained; bias-corrected anatomical images and tissue probability maps were not stored.

*Functional preprocessing.* Functional preprocessing was performed on a per-run basis. Rigid-body motion correction was first applied using SPM12 realignment, yielding six motion parameters (three translations and three rotations). Only the mean EPI image was resliced, while the original time series remained unresliced prior to normalization to minimize unnecessary interpolation. The mean EPI image was coregistered to the subject's T1-weighted image using estimate-only rigid-body coregistration. Subsequently, the original (non-resliced) functional volumes were normalized directly to MNI space using the deformation field obtained from anatomical segmentation. During normalization, SPM12 internally combined per-volume realignment parameters with the nonlinear warp, producing spatially normalized BOLD volumes without intermediate resampling. Normalized images were written at 3 mm isotropic resolution using a standard MNI bounding box. For multi-echo datasets, a single echo was selected deterministically according to a fixed echo-preference rule prior to preprocessing and processed identically to single-echo data.

*Preservation of temporal dynamics*. No slice-timing correction, spatial smoothing, temporal filtering, or ICA-based denoising was applied at this stage. These steps were intentionally omitted to preserve native temporal dynamics and inter-regional covariance prior to Koopman-based spectral analysis. Motion parameters were retained for later regression at the ROI level.

*ROI definition and time-series extraction.* Region Of Interest (ROI) time series were extracted from memory-related regions implicated in consolidation, including the hippocampus, medial prefrontal cortex, angular gyrus, and lateral temporal cortex. ROIs were defined in MNI space using atlas-based masks (BASC Cambridge atlas) and applied uniformly across datasets. For each ROI, voxel-wise BOLD signals were averaged to obtain a representative regional time series. For each subject and condition, ROI-averaged BOLD time series were extracted from memory-related regions, including the hippocampus, medial prefrontal cortex, angular gyrus, and lateral temporal cortex. At each time step t, the ROI signals were assembled into the multivariate signal vector

(1) $$\begin{array}{cc} y_{t}={\left[y_{t}^{\text{HPC}}y_{t}^{\text{mPFC}}y_{t}^{\text{AG}}y_{t}^{\text{LTC}}\right]}^{⊤}\text{,} & \end{array}$$

where each component denotes the representative signal from one ROI or ROI group. The sequence

(2) $${\left\{y_{t}\right\}}_{t=1}^{T}$$

constitutes the multivariate ROI trajectory for a given subject and condition. Koopman/DMD operators were estimated separately for each subject and condition using time-shifted snapshot pairs $\left(y_{t},y_{t+1}\right)$ constructed from this trajectory. Consequently, the reported eigenvalue-based persistence metrics characterize the latent dynamical stability of the selected memory-related network as a whole, rather than the activation magnitude or individual contribution of a single ROI. Subsequent temporal preprocessing was performed outside SPM12 using identical parameters across datasets, including regression of motion parameters, linear detrending, high-pass filtering at 0.01 Hz, and per-ROI z-scoring. This standardized ROI-level preprocessing pipeline ensured cross-dataset comparability while preserving dynamical structure relevant for spectral stability analyses. All preprocessing decisions were made prior to spectral analysis to avoid outcome-dependent bias.

### 3.3 Koopman spectral analysis and observability-aware learning

Let $y_{t}\in R^{p}$ denote the preprocessed ROI-level fMRI signal at time step t. The underlying neural state is not directly observed. We therefore define a possibly nonlinear lifting

(3) $$\begin{array}{cc} \Psi_{\theta }:R^{p}\to R^{d},z_{t}=\Psi_{\theta }\left(y_{t}\right)={\left[\psi_{1}\left(y_{t}\right)\cdots \psi_{d}\left(y_{t}\right)\right]}^{⊤}\text{.} & \end{array}$$

The finite-dimensional Koopman approximation K is learned such that the lifted coordinates evolve approximately according to

(4) $$\begin{array}{cc} z_{t+1}\approx Kz_{t} & \end{array}$$

Standard DMD is recovered as the special case in which $\Psi_{\theta }\left(y_{t}\right)=y_{t}$, whereas the learned-dictionary formulation estimates a nonlinear lifting whose coordinates evolve approximately linearly under K. In practice, infinite-dimensional Koopman operators are approximated through finite-dimensional surrogates constructed from data. Prominent approaches include Dynamic Mode Decomposition (DMD) (Schmid, 2010; Tu, 2013), Extended DMD (Williams et al., 2015), Hankel DMD (Arbabi and Mezić, 2017), and Kernel DMD (Williams et al., 2014), each differing in how observables are selected and projected. Recent work has further emphasized the importance of identifiability and observability in learned Koopman representations, particularly in the context of output-constrained lifting and invariant subspace recovery (Bartzioka and Khosravi, 2026). The observability-aware regularization adopted here builds upon this framework and adapts it to large-scale neuroimaging data.

Spectral decomposition of K yields eigenvalues $\lambda_{i}$ and corresponding spatial modes, which characterize latent dynamical components of the measured brain activity. Beyond prediction, Koopman-based models have been employed for control design, model predictive control, and state estimation (Korda and Mezić, 2018; Bevanda et al., 2021; Strässer et al., 2024), highlighting the broader systems-theoretic relevance of lifted linear representations.

*Spectral stability metrics.* Persistent consolidation-related representations can be interpreted as approximate Koopman-invariant subspaces, where dominant eigenmodes remain stable under the learned linear evolution (Brunton et al., 2016). To quantify consolidation-related persistence, we computed:

• Mean eigenvalue magnitude: $\frac{1}{N}\sum_{i=1}^{N}|\lambda_{i}|$,
• Near-unit eigenvalue count: $\#\left\{i:0.95<|\lambda_{i}|<1\right\}$,
• Pre-stabilization eigenvalue exceedance count: $\#\left\{i:|\lambda_{i}|\geq 1\right\}$.

The mean eigenvalue magnitude and near-unit count characterize persistence in the stabilized model. The exceedance count is obtained from the unconstrained preliminary DMD spectrum and is reported only as a diagnostic pre-stabilization quantity.

*Limitations of fixed dictionaries*. Standard DMD and EDMD rely on predefined observable dictionaries. However, in neuroimaging settings the relevant latent coordinates are not known a priori and may vary across tasks and subjects. Fixed dictionaries can thus either under-represent nonlinear interactions or introduce excessively high-dimensional lifts, compromising interpretability. While fully capturing nonlinear dynamics may require infinite-dimensional observables, recent theoretical results show that suitably chosen finite-dimensional Koopman-invariant subspaces can provide accurate approximations under structural assumptions on the underlying system (Fan et al., 2022). This motivates learning compact yet expressive lifted representations tailored to consolidation neural dynamics.

*Adaptive dictionary learning.* To address this limitation, we adopt a data-driven lifting approach inspired by neural dictionary learning approaches to Koopman approximation (Takeishi et al., 2017), and representation learning strategies tailored to estimation and system identification (Surana and Banaszuk, 2016). We are thus learning a nonlinear map $\Phi_{\theta }$ that lifts ROI signals into a latent space, $z_{t}=\Phi_{\theta }\left(y_{t}\right)$, where θ denotes trainable parameters. In the lifted space, dynamics evolve approximately linearly, $z_{t+1}\approx Kz_{t}$, with a linear output map ${\hat{y}}_{t}=Cz_{t}$, maintaining interpretability with respect to measured signals.

*Observability-aware extension.* Beyond predictive accuracy, we promote identifiability of lifted coordinates from measured outputs. Prior work has highlighted the importance of observability and output-consistent Koopman representations in nonlinear systems (Mesbahi et al., 2021) and output-anchored EDMD formulations (Park and Hwang, 2024). Motivated by these developments and building on an identifiability-promoting Koopman framework (Bartzioka and Khosravi, 2026), we introduce a short-horizon observability regularization to improve lifted-to-output conditioning. The matrix $C\in R^{p\times d}$ denotes the linear observation or readout matrix from the lifted coordinates to the measured ROI-level fMRI signal,

(5) $$\begin{array}{cc} {\hat{y}}_{t}=Cz_{t}\text{.} & \end{array}$$

The pair (K,C) therefore defines a linear dynamical system in the lifted observable space, with K describing the evolution of the lifted coordinates and C describing how these coordinates are expressed in the measured fMRI signals. The finite-horizon observability Gramian used in the observability-aware objective is

(6) $$\begin{array}{cc} W_{o,\tau }=\sum_{i=0}^{\tau -1}{\left(K^{i}\right)}^{⊤}C^{⊤}CK^{i}\text{.} & \end{array}$$

In standard DMD with identity observables, C may be interpreted as the identity or as a coordinate readout. In the learned-dictionary setting, C is estimated together with the lifted representation and the Koopman approximation.

Large singular values of the associated observability matrix imply that distinct latent directions remain distinguishable at the output level. We therefore introduce an observability regularization term proportional to

(7) $$\begin{array}{cc} L_{obs}=-\text{logdet}\left(W_{o,\tau }+\varepsilon I\right)\text{.} & \end{array}$$

encouraging maximally informative latent coordinates while maintaining numerical stability.

*Learning objective.* The overall learning problem jointly optimizes the lifting $\Phi_{\theta }$, linear dynamics K, output map C, and optional nonlinear reconstruction map $\Xi_{\gamma }$ by minimizing

(8) $$\begin{array}{cc} L=\lambda_{lin}∥Y-CZ_{0}{∥}_{F}^{2}+\lambda_{inv}∥Z_{1}-KZ_{0}{∥}_{F}^{2}+\lambda_{rec}∥Y-\Xi_{\gamma }\left(Z_{0}\right){∥}_{F}^{2}+\lambda_{obs}L_{obs}+\lambda_{stab}R_{stab}\left(K\right)\text{,} & \end{array}$$

where each term promotes, respectively, linear output consistency, Koopman invariance, reconstruction fidelity, lifted observability, and spectral stability. To ensure well-conditioned dynamics, spectral shrinkage or Schur-based stabilization was applied when necessary to constrain the spectral radius of K within the unit disk. This observability-aware Koopman learning framework yields a finite-dimensional, linearly evolvable, output-identifiable latent representation tailored to neuroimaging data.

**Fig. 2:**
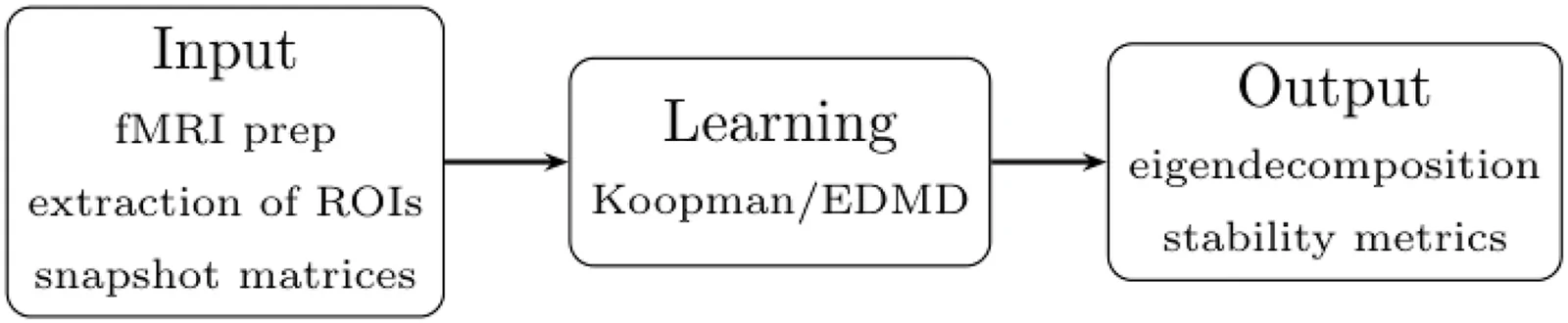
Schematic representation of the pipeline for neuroimaging data.

Full derivations and algorithmic details are provided in Appendix A–C. Fig. 2 summarizes the pipeline for analyzing fMRI neuroimaging data using the Koopman operator.

## 4 Results

Across all datasets, Koopman spectral decomposition revealed condition-dependent differences in latent dynamical stability. Early encoding or retrieval phases were characterized by broader eigenvalue dispersion, reflecting more transient and heterogeneous dynamics. In contrast, delayed or repeated retrieval conditions exhibited increased concentration of eigenvalues near the unit circle, indicating enhanced persistence of latent modes. Mean eigenvalue magnitude and near-unit eigenvalue counts varied systematically across experimental contrasts, motivating the cluster-specific analyses presented below.

### 4.1 Spectral stability metrics across consolidation stages

All spectral persistence metrics reported in the main analysis are computed after the stabilization step. In particular, after Schur retraction, the eigenvalue blocks of the learned operator are scaled to lie within the disk of radius 1−δ. Hence, the eigenvalues used for the reported stabilized Koopman spectra satisfy $\left|\lambda_{j}\right|\leq 1-\delta$ by construction, where δ>0 is the spectral stability margin defined in Appendix C.

Unconstrained DMD spectra are reported only as diagnostic outputs. All primary persistence metrics are computed from the stabilized observability-aware Koopman model, whose eigenvalues are constrained to the unit disk.

To quantify latent stability, we examined the spectrum of the learned Koopman operators for each subject and condition. Spectral decomposition yielded eigenvalues $\left\{\lambda_{i}\right\}$, whose magnitudes $\left|\lambda_{i}\right|$ provide a direct measure of temporal persistence in the lifted dynamical space. Across datasets, early encoding phases exhibited broader spectral dispersion, while delayed or repeated retrieval phases showed a shift of eigenvalues toward the unit circle. We summarize spectral behavior using three operator-level metrics:

1. Mean eigenvalue magnitude,
2. Near-unit eigenvalue count (0.95<|λ|<1),
3. Pre-stabilization eigenvalue exceedance count (|λ|≥1).

Near-unit eigenvalues correspond to slowly decaying latent representations, whereas smaller magnitudes reflect rapidly vanishing dynamics. These stability metrics operationalize cluster-specific theoretical predictions in terms of representational persistence rather than activation amplitude. The exceedance count is computed from the unconstrained preliminary DMD spectrum and is not interpreted as instability of the final Schur-stabilized operator.

To assess the statistical significance of these patterns at the subject level, permutation tests were conducted for each cluster (N=20,35,39 respectively), shuffling condition labels within subjects to generate null distributions. Effect sizes are reported as paired-sample Cohen's values $d_{z}$, which we use consistently throughout to quantify the magnitude of within-subject condition differences. Full subject-level results are presented within each cluster-specific subsection below.

### 4.2 Cluster-specific spectral signatures

We next evaluated whether the observed stability profiles aligned with the dynamical predictions derived for each consolidation cluster. To make the dataset-specific analyses interpretable without requiring the reader to consult the original studies, we first summarize the experimental design and condition labels for each dataset in Table 2.

• In Cluster 1, the relevant comparison concerns recent versus remote retrieval conditions, with resting-state data treated separately because rest may reflect a mixture of spontaneous processes rather than cued retrieval alone.
• In Cluster 2, Film indicates the encoding period during which participants viewed distressing film clips, React represents the recall/reactivation-related condition, and BI denotes the dataset-specific baseline/rest-related condition defined in the original task files.
• In Cluster 3, Internal details refer to episodic details specific to the recalled event, whereas External details refer to semantic, repeated, or generalized information.

#### 4.2.1 Cluster 1: Standard consolidation (H_1_)

Cluster 1 (dataset ds005581) evaluates the standard consolidation hypothesis, which posits that memory representations gradually stabilize over time and become supported by increasingly persistent neural dynamics. Under this framework, remote memories are expected to exhibit slower temporal decay and enhanced stability relative to recently encoded traces. At the subject level (N=20), the theory-relevant similarity judgment contrast showed a remote mean |λ|=0.9836 vs a recent mean |λ|=0.9834 in the predicted direction but was not significant ($p=0.599,d_{z}=-0.12$). All conditions exhibited uniformly high spectral persistence (E[|λ|]≈0.985), suggesting a potential ceiling effect at BOLD resolution.

Consistent with this prediction, Koopman operators identified from encoding and similarity judgment tasks exhibit a substantial concentration of eigenvalues near the unit circle. Across tasks and sessions, Cluster 1 models show high mean eigenvalue magnitudes (E[|λ|]≈0.94) and a large number of near-unit eigenvalues, indicating the presence of slowly decaying latent modes. Moreover, remote retrieval conditions consistently display greater spectral stability than recent ones, reflected in both increased mean eigenvalue magnitude and elevated counts of near-unit eigenvalues. These findings suggest that consolidation manifests as the emergence of persistent latent dynamics that support stable memory retrieval.

In contrast, resting-state conditions exhibit a different spectral pattern. Here, recent memories show slightly higher stability than remote ones, consistent with empirical evidence that resting-state activity reflects a heterogeneous mixture of spontaneous processes, including preferential replay of recently encoded material. From an operator-theoretic perspective, this results in dominant Koopman modes associated with recently active neural trajectories rather than long-term consolidated representations. Consequently, rest introduces competing dynamics that partially obscure consolidation-related signatures.

Taken together, Cluster 1 shows directionally consistent spectral patterns at both pooled and subject levels. However, subject-level effects remain small and not statistically significant, with all reported $d_{z}$ values close to zero ($-0.32\leq d_{z}\leq 0.12$, N=20), indicating that the corresponding differences are subtle at the resolution of the present data.

#### 4.2.2 Cluster 2: Episodic replay during rest (H_2_)

Cluster 2 (dataset ds003721) evaluates episodic replay-based accounts of memory consolidation, which posit that the postencoding rest period is a critical window during which neural patterns associated with encoding are reactivated and stabilized. Under this framework, retrieval-related neural dynamics during rest are expected to reflect transient reactivation processes rather than sustained persistence, producing spectral signatures characterized by broader eigenvalue dispersion and the presence of labile, short-lived modes.

Dataset ds003721 directly operationalizes this prediction. Participants underwent fMRI while watching six distressing film clips, each followed by a 5-min postencoding rest period during which consolidation-related replay was expected to occur. This encoding-rest interleaved structure aligns naturally with the core dynamical assumption of $C_{2}$: that rest intervals following encoding should exhibit spectral signatures of active but transient neural reactivation rather than long-term stable persistence.

Koopman operators trained across task conditions in this dataset revealed highly significant spectral differences. Subject-level permutation testing (N=35) showed that all pairwise task comparisons reached significance at p=0.0001 across the two primary persistence metrics. Significant condition differences were also observed for the supplementary pre-stabilization exceedance count, which is reported only as a diagnostic quantity. Specifically, mean eigenvalue magnitudes and near-unit eigenvalue counts differed substantially across conditions, with the ordering BI>React>Film observed consistently. The BI condition exhibited the highest spectral persistence (mean |λ|=0.9795, near-unit count =45.86), while the Film condition showed the lowest (mean |λ|=0.9570, near-unit count =35.71), with the React condition falling between them (mean |λ|=0.9685, near-unit count =39.69).

These differences are statistically robust. At the same time, the associated $d_{z}$ values are exceptionally large, ranging from 1.50 to 12.73, and therefore warrant careful interpretation. In the present setting, effects of this magnitude likely reflect not only consolidation-related differences, but also structural differences between task types, including run length, temporal autocorrelation, and the inherent signal complexity of film viewing, rest, and active recall. Accordingly, these results are best interpreted as strong evidence of spectral differentiation across post encoding conditions rather than as specific evidence for replay mechanisms alone.

From an operator-theoretic perspective, the lower spectral persistence observed during Film viewing relative to rest conditions is consistent with the $C_{2}$ prediction that encoding-phase neural dynamics are more transient and heterogeneous than postencoding rest dynamics. The React condition, involving active recall attempts, shows intermediate persistence, suggesting that retrieval engages dynamics that are neither as transient as encoding nor as stable as rest. This ordering is broadly consistent with the theoretical picture of episodic replay as a transient consolidation process occurring preferentially during rest.

#### 4.2.3 Cluster 3: Distributed long-term storage (H_3_)

Cluster 3 (dataset ds003511) should be interpreted as a test of representational transformation associated with distributed long-term storage accounts, rather than as a direct temporal contrast of consolidation. The external versus internal detail-related dynamics distinction separates context-rich, event-specific, episodic details from more semantic, generalized, repeated or non-event-specific autobiographical content. The present analysis therefore asks whether these two forms of recalled content exhibit different latent dynamical persistence profiles.

Subject-level permutation testing (N=39) revealed statistically significant differences between INT and EXT spectral metrics, providing the most statistically robust subject-level findings across all three clusters. EXT conditions showed significantly higher mean eigenvalue magnitude than INT conditions (EXT =0.9852, INT =0.9839; mean difference =+0.0013; p=0.017, $d_{z}=0.39$). Near-unit eigenvalue counts were also significantly higher for EXT than INT (EXT =56.87, INT =55.64; mean difference =+1.23; p=0.008, $d_{z}=0.45$). Pre-stabilization eigenvalue exceedance counts showed a trend in the opposite direction (EXT =31.79, INT =33.18; p=0.086, $d_{z}=-0.29$). Because this quantity is derived from the unconstrained preliminary DMD spectrum, it is reported only as a diagnostic and is not interpreted as instability of the final stabilized Koopman model.

External details showed greater spectral persistence than Internal details, as reflected in higher mean eigenvalue magnitude and a greater number of near-unit modes. This finding indicates that more semanticized or abstracted autobiographical content was associated with more slowly decaying latent dynamics than event-specific episodic content. It is compatible with the prediction that representational abstraction and distributed storage are associated with persistent neural dynamics. However, the comparison does not establish that External details are intrinsically more consolidated than Internal details, nor does it demonstrate that the observed difference was caused by a temporal consolidation process.

### 4.3 Cross-cluster comparison of dynamical fingerprints

To compare consolidation hypotheses at the level of latent dynamics, we report summary statistics of the Koopman spectra learned for each cluster, averaged across tasks and sessions. Table 3 presents both pooled spectral summaries and subject-level statistical results for each cluster.

**Table 3:** Pooled and subject-level statistical summary across the three clusters.

Cluster	N	$E{\left[\left|\lambda \right|\right]}_{\text{pooled}}$	Primary contrast	$p_{\text{subject}}$	$d_{z}$
$C_{1}$	20	≈0.94	similarity	0.599	−0.12
$C_{2}$	35	0.82−0.92	BI/Film/React	0.0001	1.5−12.7
$C_{3}$	39	≈0.98	EXT/INT	0.017	0.39

Subject-level permutation testing revealed different levels of evidence across clusters. Cluster $C_{3}$ provided the clearest and most theoretically interpretable subject-level support, with significant INT–EXT differences in both primary persistence metrics (mean |λ|: p=0.017, $d_{z}=0.39$; near-unit count: p=0.008, $d_{z}=0.45$; N=39). These effects were modest but consistent across metrics and aligned with H3. The supplementary pre-stabilization exceedance count did not reach significance and is not used in the primary interpretation. Cluster $C_{2}$ also showed highly significant differences across post-encoding task conditions (p=0.0001, N=35); consistent with H2 at the level of spectral differentiation, although the large effect sizes ($d_{z}$ up to 12.73) suggest that task structure contributes importantly to the observed separation. Cluster $C_{1}$ showed directionally consistent but non-significant differences (p=0.599; $d_{z}=-0.12$, N=20) in the predicted direction for H1, suggesting that the corresponding effects are subtle at the temporal and spatial resolution of the present data.

An important observation concerns the relationship between pooled and subject-level spectral estimates. For $C_{3}$, a discrepancy exists between the pooled E[|λ|] derived from aggregated snapshot matrices and the subject-level mean values of approximately 0.984. This discrepancy reflects the fact that pooled Koopman operators capture group-level temporal structure across all subjects simultaneously, whereas subject-level operators are estimated per individual and then averaged. The two quantities are not directly comparable and should be interpreted separately: pooled operators characterize the dominant dynamical structure of the group-level signal, while subject-level operators quantify individually reproducible spectral patterns. Critically, it is the subject-level analysis that provides statistically valid inference and should be treated as the primary evidence.

**Fig. 3:**
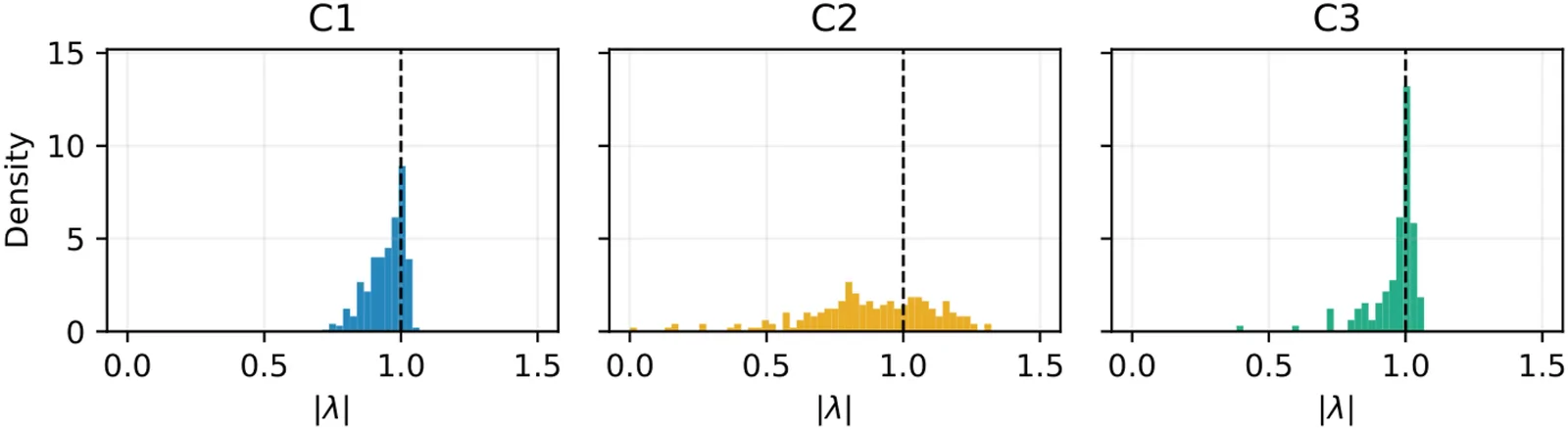
Koopman eigenvalue-magnitude distributions shown separately for each memory-consolidation cluster. The dashed line indicates the unit-circle boundary. Eigenvalues beyond the unit circle belong to the unconstrained preliminary DMD spectra and are shown only as diagnostic pre-stabilization outputs; they are not eigenvalues of the final Schur-stabilized operators used for the primary persistence interpretation.

At the pooled level, Fig. 3 shows the empirical distributions of |λ| across all trained systems. $C_{1}$ exhibits a pronounced concentration of eigenvalues near unity, indicating slowly evolving near-invariant modes consistent with progressive memory stabilization. $C_{2}$ presents a broader spectral profile with substantial mass near unity across task conditions, reflecting distributed and partially overlapping dynamical regimes. $C_{3}$ displays a narrower spectral distribution with eigenvalues concentrated well below unity at the pooled level, though as noted above this pooled estimate does not reflect the subject-level picture where INT and EXT conditions both show high spectral persistence with meaningful but small differences between them.

**Fig. 4:**
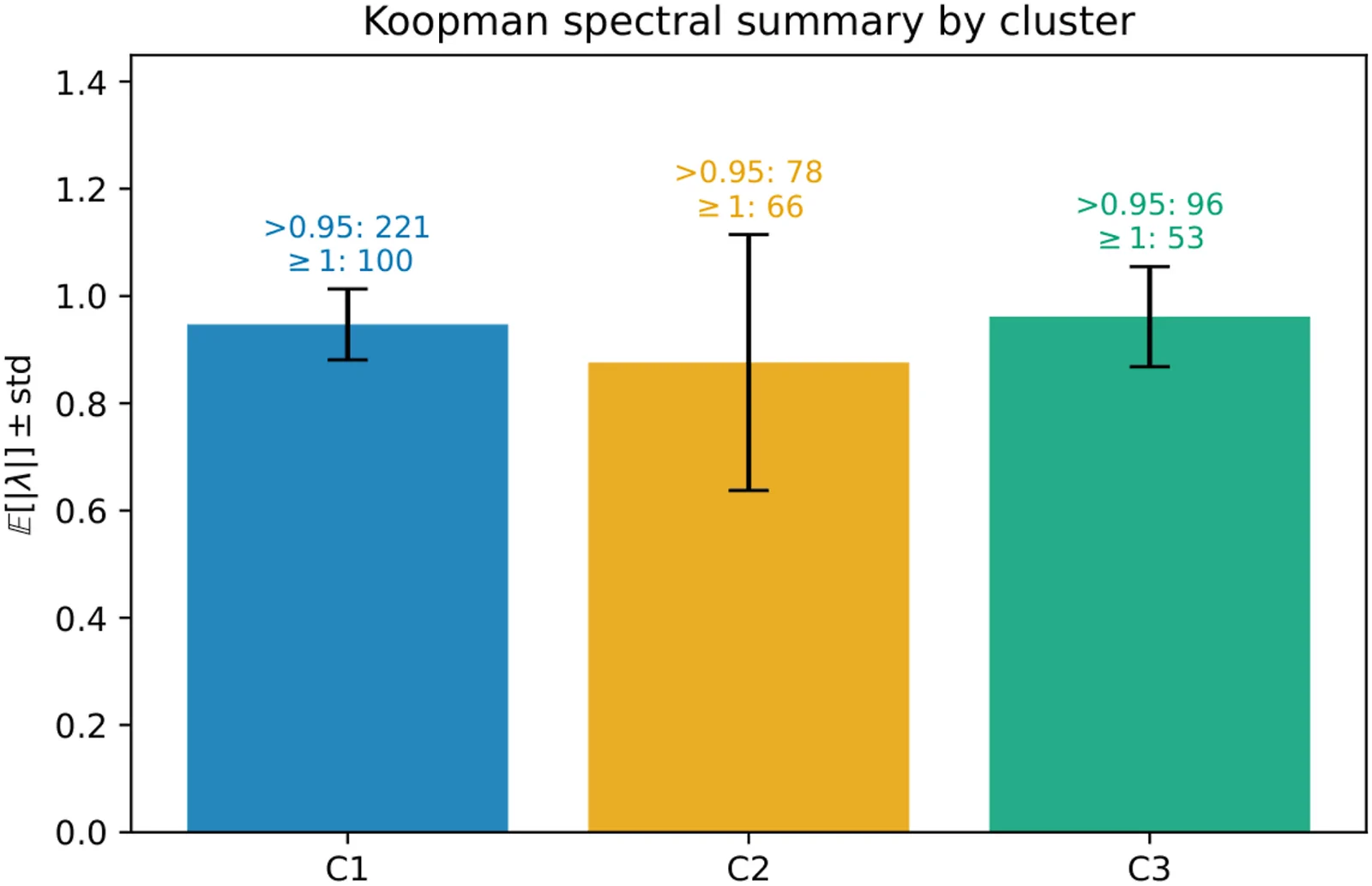
Mean and standard deviation of Koopman eigenvalue magnitudes per cluster. Error bars denote one standard deviation. Annotations indicate the number of near-unit modes and the pre-stabilization eigenvalue exceedance count. The latter is obtained from the unconstrained preliminary DMD spectra and is shown only as a diagnostic quantity.

Fig. 4 summarizes mean and standard deviation of |λ| per cluster, together with near-unit counts and diagnostic pre-stabilization eigenvalue exceedance counts. Across clusters $C_{1}$ and $C_{2}$ exhibit substantially higher pooled spectral mass near unity than $C_{3}$, though this ordering reverses at the subject level where $C_{3}$ provides the most statistically robust evidence. This divergence between pooled and subject-level results underscores the importance of moving beyond aggregated spectral summaries toward statistically grounded individual-level inference.

Taken together, the three datasets exhibited distinct latent dynamical fingerprints relative to the proposed hypotheses.

• **H1 (standard consolidation):** directionally consistent but small increases in persistence for remote relative to recent conditions.
• **H2 (episodic replay during rest):** strong spectral differentiation across post-encoding states, with interpretation tempered by task-structural contributions.
• **H3 (distributed long-term storage):** the clearest subject-level evidence, with EXT showing greater persistence than INT across multiple spectral metrics.

These considerations motivate the following proposition, which formalizes the connection between Koopman spectral properties and memory persistence.

**Persistent memory representations** correspond to Koopman eigenmodes with eigenvalues of magnitude close to unity. Such near-invariant modes encode latent brain dynamics that evolve slowly over time, enabling long-term retention of information.

Distributed long-term storage provides the clearest subject-level evidence for this principle, while standard consolidation shows directionally consistent but non-significant trends and episodic replay shows strong spectral differentiation whose interpretation is tempered by task-structural contributions.

## 5 Discussion

In this paper, we introduced a biologically informed Koopman operator framework to identify latent dynamical fingerprints of human memory consolidation directly from fMRI time series. By reformulating consolidation theories in terms of predicted stability structure, we translated conceptual differences between memory models into measurable spectral signatures. Across datasets, our results show that consolidation accounts can be differentiated at the level of latent dynamical stability rather than activation magnitude alone. Importantly, this conclusion is supported not only by pooled spectral summaries, but also by subject-level statistical analyses, which revealed that the principal differences in eigenvalue magnitude were expressed consistently across individuals.

The network-level persistence findings should be interpreted in light of the distinct theoretical roles of the included ROIs. Under standard consolidation, remote retrieval is expected to rely increasingly on neocortical representations, particularly in medial prefrontal and lateral temporal regions, whereas hippocampal involvement is generally expected to be stronger for recent or context-rich episodic retrieval. However, because the primary eigenvalue-based metrics are computed from the joint multivariate ROI trajectory, they characterize the dynamics of the selected network as a whole and do not identify the contribution of an individual ROI. Accordingly, a network-level spectral difference should not be attributed to a specific region unless this interpretation is supported by an additional ROI-specific or spatial-mode analysis.

### 5.1 Persistent eigenmodes as signatures of stabilized memory traces

Across datasets, delayed retrieval and repeated retrieval conditions were associated with an increased concentration of eigenvalues near the unit circle, that is, with magnitudes approaching one. In the Koopman framework, eigenvalue magnitude quantifies temporal persistence: eigenvalues near unity correspond to slowly decaying latent modes, whereas smaller magnitudes reflect more transient dynamics.

The standard-consolidation interpretation requires consideration of the distinct theoretical roles of the included ROIs. Remote retrieval is expected to rely increasingly on neocortical representations, particularly in medial prefrontal and lateral temporal regions, whereas hippocampal involvement is generally expected to be stronger for recent or context-rich episodic retrieval.

The hippocampal pattern is theoretically more nuanced. Under standard consolidation, hippocampal dependence may decrease as memories become remote. Under Multiple Trace Theory or transformation-oriented accounts, however, the hippocampus may remain engaged when retrieval involves vivid, event-specific, or context-rich episodic detail.

Because the primary eigenvalue-based metrics are computed from the joint multivariate ROI trajectory, an observed network-level persistence difference cannot be attributed to an individual ROI without an additional ROI-specific or spatial-mode analysis. The findings should therefore be interpreted as network-level evidence compatible with these ROI-specific theoretical expectations, rather than as a uniform increase in persistence across both hippocampal and neocortical regions.

### 5.2 Replay-related dynamics and distributed persistence

The contrast between Clusters $C_{2}$ and $C_{3}$ helps to clarify an important distinction between replay-based and distributed-storage accounts of consolidation. In Cluster $C_{2}$, corresponding to episodic replay during rest (ds003721), the main signature is state-dependent spectral separation across post-encoding conditions. This is compatible with replay-based views, but the magnitude of the separation suggests that the effect reflects both consolidation-related dynamics and broader task structure. In other words, $C_{2}$ supports the presence of distinct post-encoding dynamical regimes, but it does not isolate replay as their unique source.

The findings for Cluster 3, corresponding to distributed long-term storage (ds003511), should be interpreted cautiously. The External–Internal contrast does not constitute a direct recent–remote comparison and therefore does not provide a direct temporal test of consolidation. Rather, it supports a narrower representational finding: within autobiographical recall, more semanticized, generalized, or abstracted content was associated with greater latent persistence than context-rich, event-specific episodic content.

This pattern is compatible with transformation-oriented and distributed-storage accounts, including Multiple Trace Theory and Complementary Learning Systems perspectives, which propose that repeated retrieval and abstraction may support increasingly distributed representations. Nevertheless, the present analysis cannot establish that External details are more consolidated than Internal details, that episodic memories do not consolidate, or that semantic information uniquely indexes consolidation. The result should therefore be regarded as evidence of a difference in the latent dynamical organization of episodic and more semanticized autobiographical content, rather than as direct evidence of the temporal consolidation process itself.

### 5.3 Operator-theoretic interpretation of consolidation

The main conceptual contribution of this work is the reframing of memory consolidation as a problem of latent dynamical stability. Rather than assessing theories only through static contrasts in activation amplitude, we evaluate how neural representations evolve and persist over time in a learned lifted state space. In the Koopman representation: eigenvalues quantify representational persistence, eigenfunctions encode invariant temporal transitions and spatial modes localize dynamical patterns across brain regions.

By linking these spectral components to theory-driven predictions, we establish a principled bridge between systems neuroscience and operator theory. Within this perspective, consolidation is not merely a redistribution of activation across regions, but a reorganization of the temporal stability structure of latent neural dynamics.

Moreover, our observability-aware extension helps ensure that the learned latent coordinates remain identifiable from measured outputs, thereby reducing the risk of trivial or non-informative lifted representations. This is especially relevant in neuroimaging settings, where latent-state interpretability is essential if spectral quantities are to be related meaningfully to cognitive theory.

### 5.4 Methodological implications

Methodologically, this work shows that operator-theoretic tools can extract biologically meaningful dynamical signatures from fMRI data without requiring detailed mechanistic modeling. The proposed framework is model-agnostic, applicable across datasets, interpretable in terms of stability theory and compatible with large-scale neuroimaging data.

An important additional implication of the present results is that subject-level statistical analysis can be integrated naturally into Koopman-based neuroimaging pipelines. The fact that the principal spectral differences were reflected at the participant level strengthens the case for treating Koopman-derived persistence metrics as experimentally meaningful observables rather than descriptive summaries of pooled data alone. In this sense, the framework offers a complementary perspective to traditional Generalized Linear Model (GLM)-based analyses by focusing on latent dynamical organization rather than region-wise activation amplitude.

### 5.5 Limitations

Several limitations should be acknowledged. First, BOLD signals provide an indirect measure of neural activity and have limited temporal resolution, so rapid synaptic-scale consolidation processes may not be fully resolved. Second, finite-dimensional Koopman approximations assume approximately linear evolution in the lifted space; although effective in practice, this remains an approximation to potentially richer nonlinear dynamics. Third, while the subject-level analyses increased confidence in the robustness of the main findings, inter-subject variability remained non-negligible, particularly in the replay-related dataset. This suggests that future work should consider hierarchical or mixed-effects operator-theoretic models that capture both shared and participant-specific dynamical structure more explicitly. Finally, our conclusions concern stability fingerprints inferred from available task designs and timescales, rather than exhaustive validation of any single biological consolidation theory.

### 5.6 Future directions

Several extensions follow naturally from this work. Application to higher-temporal-resolution modalities such as MEG or intracranial EEG, cross-modal validation of persistence signatures, hierarchical subject-level operator models, task-general operator learning across cognitive domains and longitudinal designs tracking consolidation over days or weeks. More broadly, operator-theoretic spectral analysis may provide a generalizable framework for studying latent dynamical stability across cognitive systems beyond memory consolidation.

## 6 Conclusion

By translating memory consolidation theories into predictions about latent dynamical stability and evaluating these predictions through Koopman spectral analysis, we showed that memory-related conditions can be differentiated by their persistence structure in lifted latent space. Across datasets, delayed or repeated retrieval conditions generally exhibited greater spectral persistence than early encoding-related conditions, although the strength and interpretability of this pattern differed across theoretical clusters. Among the three analyses, Cluster 3 yielded the clearest statistically reliable subject-level contrast. However, the comparison between Internal and External autobiographical details does not constitute a direct temporal test of consolidation. Instead, more semanticized or abstracted content was associated with greater latent persistence than event-specific episodic content. This finding is compatible with transformation-oriented and distributed-storage accounts, but it does not establish that External details are intrinsically more consolidated than Internal details or that the observed difference was produced by a temporal consolidation process. Accordingly, the Cluster 3 result is interpreted as an indirect representational finding concerning latent persistence, rather than as direct validation of a specific temporal consolidation mechanism. Replay-related conditions showed marked spectral differentiation across task states, but these effects should be interpreted cautiously because task-structural factors likely contribute to the observed separation. Standard consolidation displayed directionally consistent but non-significant subject-level trends, suggesting that any corresponding effects may be subtle at BOLD resolution. Taken together, these findings establish operator-theoretic analysis as a principled and biologically interpretable framework for comparing consolidation accounts through latent dynamical signatures, while also highlighting the importance of subject-level inference and careful interpretation of pooled spectral summaries.

## CRediT authorship contribution statement

**Maria Bartzioka:** Conceptualization, Formal analysis, Methodology, Software, Visualization, Writing – original draft. **Mohammad Khosravi:** Supervision, Visualization, Writing – review & editing.

## Data and code availability statement

All neuroimaging datasets analyzed in this study were obtained from publicly available repositories adhering to the Brain Imaging Data Structure (BIDS) standard and were downloaded from OpenNeuro (https://openneuro.org). Datasets were selected based on their alignment with the consolidation clusters described above, together with task metadata completeness, preprocessing compatibility, and sufficient sample size. Raw imaging data and metadata were retrieved in BIDS format to support standardized preprocessing and reproducibility.

Preprocessing was performed using SPM12 (Wellcome Centre for Human Neuroimaging, London, UK), followed by time-series extraction and Koopman spectral analysis using custom MATLAB and Python scripts. All analysis scripts are publicly available at https://github.com/mbartzioka/koopman-for-memory-consolidation, including preprocessing pipelines, ROI extraction procedures, Koopman operator estimation routines, and documentation needed to reproduce the reported spectral metrics and figures.

No new data were collected for this study, and all analyses were conducted on de-identified, publicly available datasets in accordance with the original ethical approvals obtained by the respective study authors.

## Funding

This research did not receive any specific grant from funding agencies in the public, commercial, or not-for-profit sectors.

## Declaration of competing interests

The authors declare that they have no known competing financial interests or personal relationships that could have appeared to influence the work reported in this paper.

## Data Availability

The data used were open source.

## Appendix A Nonlinear observability and lifted factorization

Consider a nonlinear continuous-time system

$$\dot{x}=f\left(x\right),y=h\left(x\right)\text{,}$$

with $x\in R^{n}$ and $y\in R^{p}$.

### Appendix A_1_. Lie derivatives and local observability

The Lie derivatives of each output component $h_{i}$ along the vector field f are defined recursively as

$$\begin{array}{c} L_{f}^{0}h_{i}:=h_{i}\text{,}\\ L_{f}^{k}h_{i}\left(x\right):=\nabla \left(L_{f}^{k-1}h_{i}\right){\left(x\right)}^{⊤}f\left(x\right)\text{.} \end{array}$$

The local nonlinear observability map up to order q is

$$O_{q}\left(x\right)=\left[\begin{array}{c} h\left(x\right)\\ L_{f}h\left(x\right)\\ \vdots \\ L_{f}^{q}h\left(x\right) \end{array}\right]\text{.}$$

The system is locally weakly observable at $x^{⋆}$ if

$$\text{rank}\left(\frac{\partial O_{q}}{\partial x}\left(x^{⋆}\right)\right)=n$$

for some finite q.

### Appendix A_2._ Factorization through lifted coordinates

Let a differentiable lifting $\Psi :R^{n}\to R^{N}$ define lifted coordinates z=Ψ(x), with linear lifted dynamics

$$\dot{z}=Kz,\hat{y}=Cz\text{.}$$

Stacking derivatives of the predicted output yields

$$\frac{\partial }{\partial x}\left[\begin{array}{c} h\left(x\right)\\ \dot{h}\left(x\right)\\ \vdots \\ h^{\left(q\right)}\left(x\right) \end{array}\right]=\left[\begin{array}{c} C\\ CK\\ \vdots \\ CK^{q} \end{array}\right]J_{\Psi }\left(x\right)\text{,}$$

where $J_{\Psi }\left(x\right)$ is the Jacobian of the lifting.

This establishes a factorization:

$$\begin{array}{c} \text{Nonlinear}\;\text{observability}=\\ \text{Linear}\;\text{lifted}\;\text{observability}\times \text{Jacobian}\;\text{of}\;\text{lifting.} \end{array}$$

Thus, if $J_{\Psi }\left(x\right)$ is locally injective and the lifted observability block

$$O_{\tau }={\left[\begin{array}{cccc} C^{⊤} & {\left(CK\right)}^{⊤} & \ldots & {\left(CK^{\tau -1}\right)}^{⊤} \end{array}\right]}^{⊤}$$

has full column rank, output-level separability in the lift implies local observability in the original nonlinear system.

## Appendix B Lifted observability Gramian and volume interpretation

Over a finite horizon τ, the lifted observability Gramian is defined as

$$W_{o,\tau }=\sum_{t=0}^{\tau -1}{\left(K^{t}\right)}^{⊤}C^{⊤}CK^{t}\text{.}$$

### Appendix B_1_. Volume interpretation

Let $\sigma_{1},\ldots ,\sigma_{N}$ denote the singular values of the lifted observability matrix $O_{\tau }$. The volume of the transformed unit ball satisfies

$${\text{vol}}_{N}\left(O_{\tau }B_{2}^{N}\right)={\text{vol}}_{N}\left(B_{2}^{N}\right)\prod_{i=1}^{N}\sigma_{i}={\text{vol}}_{N}\left(B_{2}^{N}\right)\sqrt{\det\;W_{o,\tau }}\text{.}$$

Therefore,

$$\frac{1}{2}\text{logdet}W_{o,\tau }$$

measures log-volume expansion and quantifies distinguishability of latent directions.

### Appendix B_2_. Numerical stabilization

To avoid singularity and improve conditioning, we evaluate

$$\text{logdet}\left(W_{o,\tau }+\varepsilon I\right)$$

using Cholesky factorization:

$$W_{o,\tau }+\varepsilon I=LL^{⊤},\text{logdet}=2\sum_{i}\log\;L_{ii}\text{.}$$

This yields stable and efficient computation.

## Appendix C Optimization details and stability enforcement

### Appendix C_1_. Learning problem

Given output data ${\left\{y_{t}\right\}}_{t=0}^{T}$, parameters (θ,K,C,γ) are obtained via

$$\left(\hat{\theta },\hat{K},\hat{C},\hat{\gamma }\right)=\mathrm{argmin}L\left(\theta ,K,C,\gamma \right)\text{,}$$

where the loss function is

$$L=\lambda_{inv}∥Z_{1}-KZ_{0}{∥}_{F}^{2}+\lambda_{lin}∥Y-CZ_{0}{∥}_{F}^{2}+\lambda_{rec}∥Y-\Xi_{\gamma }\left(Z_{0}\right){∥}_{F}^{2}+\lambda_{obs}\left(-\text{logdet}\left(W_{o,\tau }+\varepsilon I\right)\right)+\lambda_{stab}R_{stab}\left(K\right)\text{.}$$

### Appendix C_2_. Stability enforcement

Let δ∈(0,1) denote a prescribed positive spectral stability margin, and define the maximum admissible spectral radius as

$$r_{\max}=1-\delta \text{.}$$

*Spectral shrinkage.* If ρ(K)>1−δ, rescale:

$$K\leftarrow \frac{1-\delta }{\rho \left(K\right)}K\text{.}$$

*Schur retraction.* Compute real Schur decomposition $K=QUQ^{⊤}$, scale eigenvalue blocks to lie within radius 1−δ, and reconstruct

$$\tilde{K}=Q\tilde{U}Q^{⊤}\text{.}$$

During Schur retraction, the eigenvalue blocks of the learned operator are rescaled, when necessary, so that the stabilized operator satisfies

$$\rho \left(K\right)\leq r_{\max}=1-\delta \text{.}$$

Consequently, every eigenvalue of the stabilized operator satisfies

$$\left|\lambda_{j}\right|\leq 1-\delta \text{.}$$

### Appendix C_3_. Training stabilization

Practical measures include:

• Short prediction horizons (τ∈[10,20]),
• Mild Tikhonov regularization on C,
• Feature whitening of lifted coordinates,
• Balanced loss weights to maintain comparable magnitudes of invariance and observability terms.
